# Development of Endogenous Protein Probes for Characterizing Surface Proteins and Cellular Interactors of Extracellular Vesicles

**DOI:** 10.1002/advs.202511414

**Published:** 2025-12-12

**Authors:** Wenyi Zheng, Metoboroghene Mowoe, Wenqing Hou, Daniel W. Hagey, Koshi Imami, Samir EL Andaloussi

**Affiliations:** ^1^ College of Pharmacy Chongqing Medical University Chongqing 400016 China; ^2^ Division of Biomolecular and Cellular Medicine Department of Laboratory Medicine Karolinska Institutet Huddinge Stockholm 14152 Sweden; ^3^ Department of Cellular Therapy and Allogeneic Stem Cell Transplantation (CAST) Karolinska University Hospital Huddinge Stockholm 14186 Sweden; ^4^ Karolinska ATMP Center Karolinska Institutet Huddinge Stockholm 14152 Sweden; ^5^ RIKEN Center for Integrative Medical Sciences Yokohama Kanagawa 230‐0045 Japan

**Keywords:** APEX2, extracellular vesicles, protein corona, proximity labeling, receptor

## Abstract

Extracellular vesicle (EV) surface proteins, derived from producer cells and their surrounding environment, represent a valuable source of biomarkers and participate in a plethora of biological functions, including intercellular communication. However, current methods struggle to distinguish core EV surface proteins from adsorbed corona proteins or map the EV‐cell interplay. Here, a genetically encoded proximity labelling probe is presented that displays engineered ascorbate peroxidase, APEX2, on the surface of EVs via fusion to EV‐sorting scaffold proteins. This enables the biotinylation of producer‐cell‐derived surface proteins, corona proteins, and interactors in vitro. After the enrichment by streptavidin bead pulldowns, subpopulation‐specific, biotinylated surfaceome and interactome are comprehensively characterized using mass spectrometry‐based proteomics. Thus, a genetic tool is introduced for the high‐fidelity mapping of the surfaceome and cellular interactome of EVs in vitro. This approach offers a robust framework for dissecting EV biology and has broad applications in biomarker discovery and EV‐based therapeutics.

## Graphical abstract

The proximity labeling enzyme APEX2 is displayed on extracellular vesicle (EV) surfaces via genetic fusion with EV‐sorting scaffolds, enabling in situ biotinylation of native surface proteins, adsorbed corona components, and interacting cellular proteins. Streptavidin enrichment coupled with mass spectrometry reveals subpopulation‐specific surfaceome and interactome, providing a precise platform for biomarker discovery and mechanistic dissection of EV‐mediated communication.

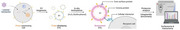

## 1 Introduction

Extracellular vesicles (EVs) are cell‐derived particles with a plethora of physiological and pathological roles,^[^ ^1^ ^]^ though the mechanisms by which they act are yet to be fully elucidated.^[^ ^2^, ^3^ ^]^ The EV surface is composed of core components from producer cells and a corona that spontaneously forms upon exposure to the extracellular environment.^[^ ^4^ ^]^ Deciphering these molecular signatures is necessary not only to reveal their mechanisms of action but also to identify effective diagnostic biomarkers.^[^ ^5^, ^6^ ^]^ Furthermore, these surface proteins collectively guide EVs toward target cells to mediate intercellular molecule exchange and contact‐dependent signaling.^[^ ^4^, ^7^, ^8^, ^9^, ^10^, ^11^, ^12^, ^13^, ^14^, ^15^, ^16^, ^17^ ^]^ Thus, mapping the repertoire of surface proteins and interacting proteins of EVs may provide deeper insight into their biological, diagnostic, and therapeutic roles.

Despite the use of state‐of‐art purification methods, most commonly differential centrifugation, EV preparations often contain considerable levels of non‐EV proteins, such as albumin and lipoproteins,^[^ ^18^, ^19^, ^20^ ^]^thereby limiting the detection of less‐abundant yet informative proteins. Moreover, the shear stress caused by sample centrifugation can lead to the dissociation and loss of EV corona proteins.^[^ ^21^ ^]^ Lastly, though many methods are available for tracking EV tropism through measuring cellular uptake,^[^ ^22^ ^]^ there is a lack of specific tools to map the cellular interactors. Therefore, an approach to characterize core surface proteins and corona proteins of EVs as well as to map interactors on recipient cells would be of broad utility.

Proximity labeling involves the use of engineered enzymes, such as peroxidases or biotin ligases, to generate highly reactive, short‐lived species that covalently tag neighboring molecules.^[^ ^23^ ^]^ Indeed, proximity labeling enzymes have been tethered to EV surfaces to map the surfaceome and cellular interactome. They are either chemically conjugated to lipid anchors/lectins, for tethering with pre‐isolated EVs,^[^ ^24^ ^]^ or genetically fused to the C1C2 domain that binds to phosphatidylserine lipids upon EV biogenesis.^[^ ^25^ ^]^ However, these approaches are limited by the dissociation of proximity labeling enzyme from EVs^[^ ^26^ ^]^, the purity of EV preparations, suboptimal catalytic activity of proximity labeling enzyme,^[^ ^23^ ^]^ and the luminal expression of phosphatidylserine.^[^ ^27^ ^]^

**Figure 1:**
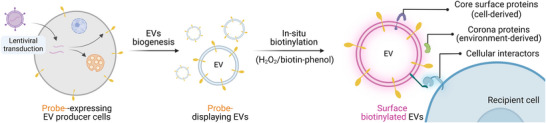
Development of protein probes for the analysis of vesicle surface proteins and interactors. EV producer cells are genetically engineered to achieve endogenous surface display of the proximity labeling enzyme APEX2 on EVs. Then, APEX2 catalyzes the biotinylation of surface proteins, including cell‐derived core components and environment‐derived corona components, as well as interactors on recipient cells.

To date, APEX2, an engineered ascorbate peroxidase, is the most active proximity labeling enzyme known.^[^ ^23^ ^]^ Using hydrogen peroxide as an electron donor, APEX2 converts the substrate biotin‐phenol to biotin‐phenoxyl radicals for the biotinylation of APEX2‐proximal proteins. Subsequently, the biotinylated proteins can be enriched through streptavidin affinity purification and annotated using mass spectrometry (MS) or other proteomic techniques. In this study, we build an endogenous protein probe leveraging APEX2 and EV‐sorting scaffold proteins, so that EV surfaces are functionalized with APEX2 for mapping surface proximal proteins, including core vesicle surface proteins, corona proteins as well as cellular interactors in vitro (**Figure** **1**).

## 2 Results

### 2.1 CD63 and TSPAN2 Support APEX2 Surface Display on EVs

**Figure 2:**
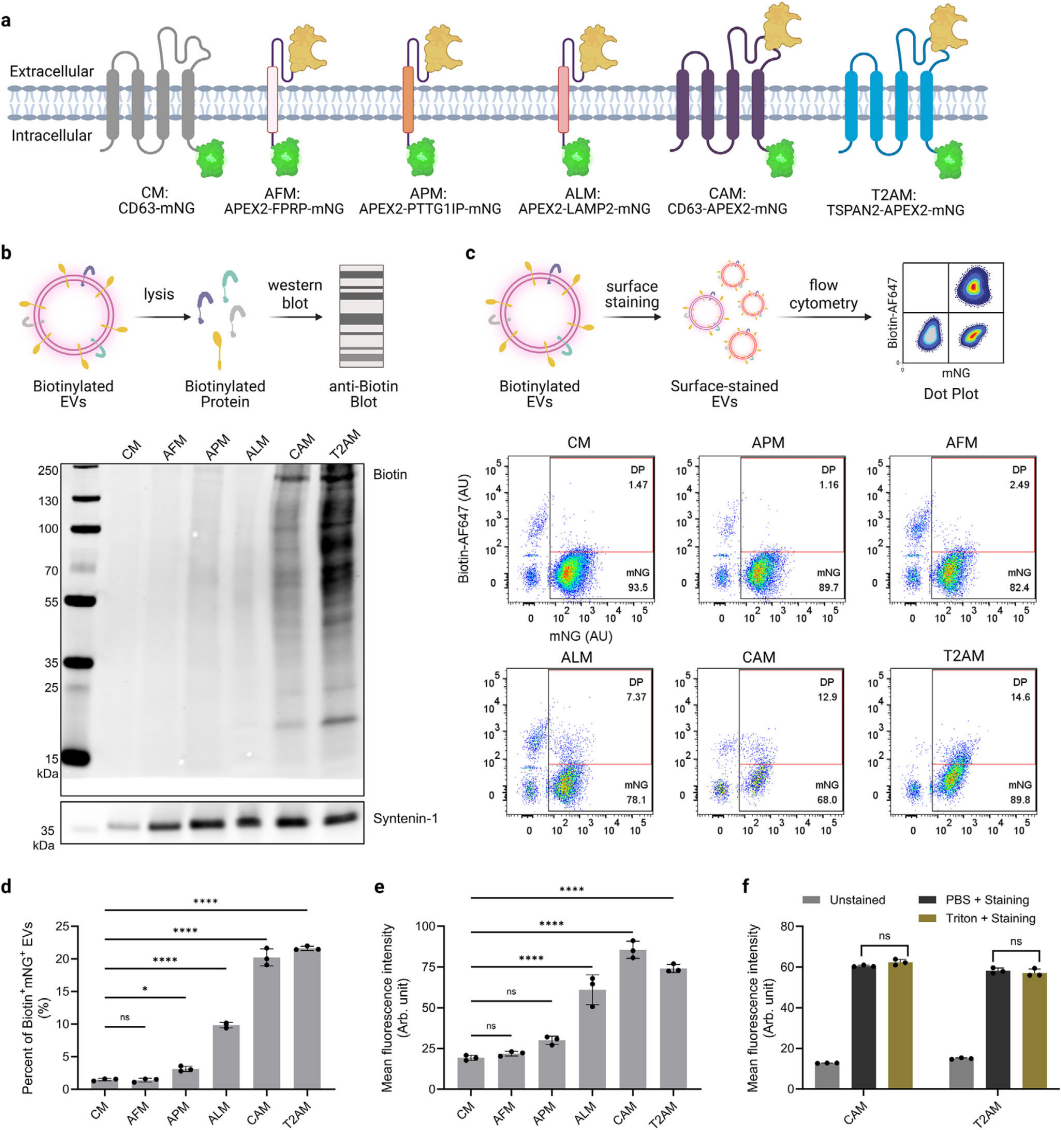
Comparing scaffold proteins for APEX2 surface display. a) Construct design for the surface display of APEX2 on EVs. b) Validation of biotinylated proteins in EV lysates using western blot. Syntenin‐1 serves as the loading control. c) Analysis of vesicle surface APEX2 activity. Biotinylated EVs were stained with streptavidin‐AF647 conjugates and analyzed using imaging flow cytometry. d) Percentage of double position (AF647^+^mNG^+^) vesicles in total engineered EVs (mNG^+^). e) Mean AF647 fluorescence intensity of mNG^+^ EVs. f) Biotinylated EVs were permeabilized using 0.001% Triton to allow dye access to intravesicular biotin and stained with streptavidin conjugates. Mean AF647 fluorescence intensity of mNG^+^ EVs was presented. In (d‐f), results are presented as mean ± standard deviation. One‐way ANOVA. ns: non‐significant; ^*^: p ≤ 0.05; ^****^: p ≤ 0.0001.

In previous studies, APEX2 has been fused with domains‐of‐interest to realize site‐restricted expression in cells; however, to the best of our knowledge, vesicle surface display of APEX2 has yet to be reported. Here, we first designed engineering strategies for displaying APEX2 on EV surfaces (**Figure** **2**a). The extracellular termini of single‐pass transmembrane proteins, such as FPRP (prostaglandin F2 receptor negative regulator), LAMP2 (lysosome‐associated membrane glycoprotein 2), and PTTG1IP (pituitary tumor‐transforming gene 1 protein‐interacting protein), have been used to display cargo peptides on EV surfaces^[^ ^28^, ^29^, ^30^ ^]^; thus, these proteins were tested in this study. In addition, since the N‐ and C‐ termini of APEX2 are in proximity,^[^ ^31^ ^]^ we assume that inserting APEX2 into the extracellular loop of EV‐sorting tetraspanin proteins (e.g., CD63^[^ ^32^ ^]^ and TSPAN2^[^ ^33^ ^]^) would not severely compromise protein folding and vesicle biogenesis. Wild‐type CD63 without APEX2 was used as the negative control. Lastly, the mNeonGreen (mNG) reporter protein was terminally fused to these constructs for intravesicular loading, to enable the detection of engineered cells and EVs. Thereafter, the six constructs were compared for their efficiency in the surface display of APEX2.

Human kidney embryonic (HEK‐293T) cells were transduced with lentiviral vectors encoding the six constructs and selected using puromycin to establish stable cell lines. At first, we examined the expression levels and activity of APEX2 on cells before collecting EVs for further analysis. Cells were biotinylated by adding biotin‐phenol and hydrogen peroxide and stained with AF647‐labeled streptavidin conjugates (Figure S1a, Supporting Information). Flow cytometry analysis shows that almost all cells, except those in the negative control (CD63‐mNG, CM) group, were double positive for mNG and surface biotin, indicating effective construct expression and APEX2 activity (Figure S1b, Supporting Information).

Next, we harvested small EVs from the cell lines using tangential flow filtration and spin filtration. The EV preparations had a median diameter ≈120 nm (Figure S2a, Supporting Information) and were negative for the exclusion marker calnexin (Figure S2b, Supporting Information). Subsequently, we examined APEX2 activity at the vesicular level. In the first method, EVs were biotinylated and lysed for biotinylated proteins via western blotting (Figure 2b). TSPAN2‐APEX2‐mNG (T2AM) exhibited the highest levels of biotin, followed by CD63‐APEX2‐mNG (CAM), APEX2‐PTTPIP‐mNG (APM), and APEX2‐LAMP2‐mNG (ALM); however, APEX2‐FPRP‐mNG (AFM) had biotinylation levels comparable to that of the negative control. In the second method, since streptavidin cannot penetrate vesicle membrane, we specifically measured surface biotin on intact EVs by imaging flow cytometry using streptavidin–AF647 fluorescence as the readout (Figure 2c). A substantial number of mNG^−^/AF647⁺ events were detected, likely representing streptavidin‐AF647 dye aggregates rather than true biotinylated EVs, as similar signals were observed in PBS‐only controls (Figure S3, Supporting Information). Therefore, we focused our analysis on APEX2 activity in mNG⁺ EVs. Aligning with the results from western blotting, T2AM and CAM EVs had the highest levels of surface biotin, with ≈20% of mNG‐positive vesicles being biotin‐positive (Figure 2d) and a 3.5‐fold increase in mean AF647 fluorescence intensity over the negative control (Figure 2e).

Because biotin‐phenol and hydrogen peroxide are membrane‐permeable, we considered the possibility that APEX2 could reside inside EVs and catalyze biotinylation of intravesicular cargos. To address this, we compared streptavidin staining intensities of biotinylated EVs with or without mild permeabilization (0.001% Triton X‐100), which allows membrane penetration of streptavidin while preserving vesicle structure. Notably, permeabilization produced no appreciable change in streptavidin staining for CAM and T2AM EVs (Figure 2f), suggesting negligible intravesicular APEX2 activity. Collectively, these results demonstrate that CD63 and TSPAN2 are suitable for displaying APEX2 on the EV surface, enabling efficient and specific surface biotinylation.

### 2.2 APEX2 Surface Labeling Identifies EV Core Surface Proteins

**Figure 3:**
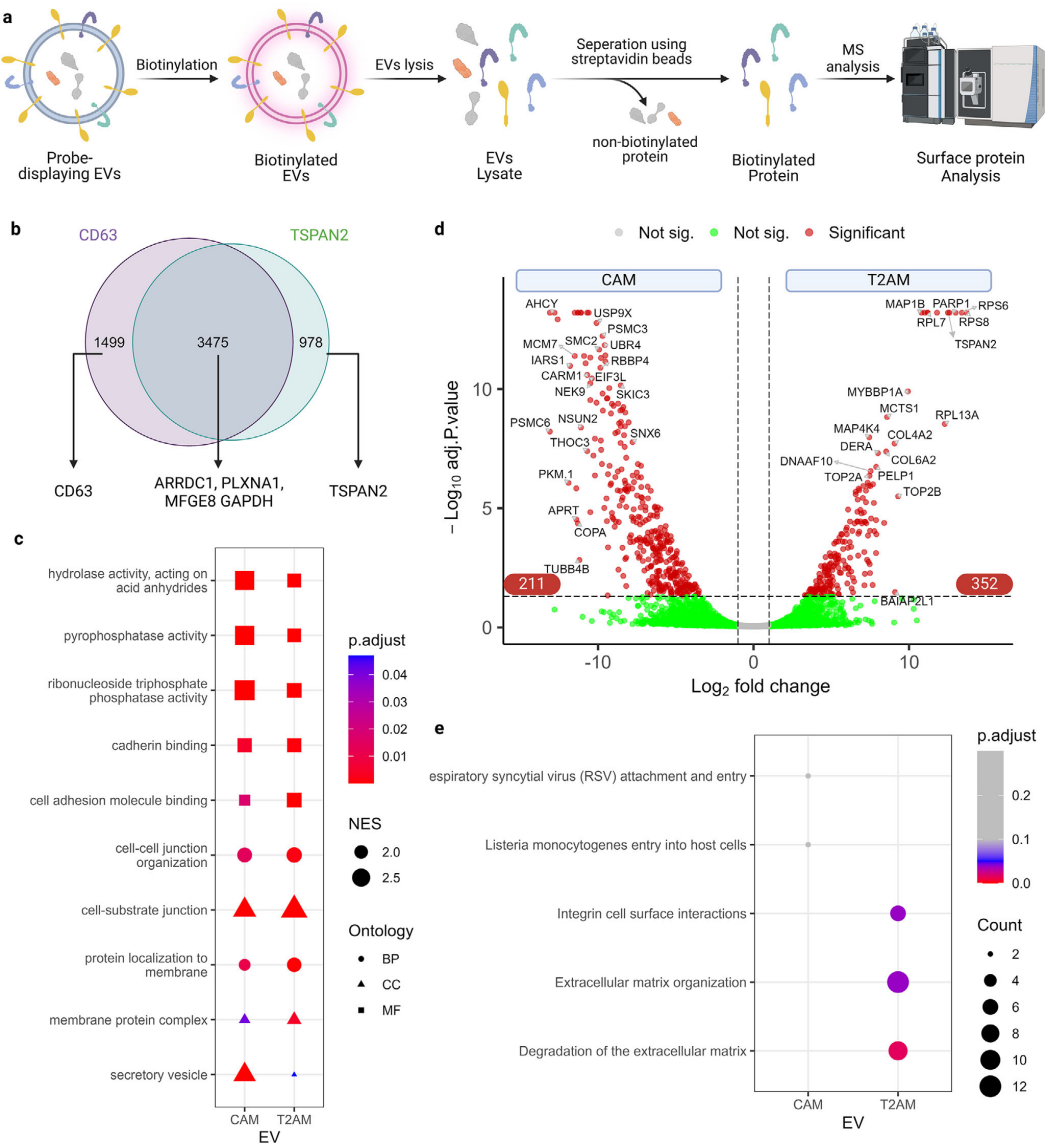
Analysis of vesicle core surface proteome. a) Probe‐displaying EVs were biotinylated in phosphate buffers containing biotin‐phenol and hydrogen peroxide and lysed to release contents. Then, biotinylated proteins were enriched using streptavidin beads and analyzed using mass spectrometry. b) Venn diagram of biotinylated proteins in CAM and T2AM EVs. Some proteins‐of‐interest were listed below. c) Gene set enrichment analysis results using the gene ontology database. NES: net enrichment score; BC: biological process; CC: cellular component; MF: molecular functions. d) Volcano plot of biotinylated proteins in CAM and T2AM EVs. P ≤ 0.05 and fold‐change ≥ 2 was considered significant. e) Pathway analysis of proteins enriched for CAM and T2AM EVs using the Reactome database.

Due to their greater efficiency in the surface display of APEX2, CD63 and TSPAN2 were used for probe construction to decipher the EV subpopulation‐specific surfaceome. To analyze core surface proteins, EVs were produced from stable cells cultured in plasma‐free Opti‐MEM media and biotinylated in phosphate buffers (**Figure** **3**a). Following the removal of unreacted biotin‐phenol using size exclusion chromatography columns, EVs were lysed to release protein contents. Afterward, streptavidin beads were applied to pull down biotinylated proteins for S7MS analysis using label‐free quantification techniques. Technical negative controls were included by omitting biotin‐phenol in the biotinylation step.

Many proteins were identified in the negative controls (Figure S4a, Supporting Information), which might be due to non‐specific protein binding to streptavidin beads. Thus, we filtered out non‐specific proteins by applying background correction against the non‐biotinylated negative controls. As proof‐of‐success, the filtering kept CD63 and TSPAN2 but excluded Syntenin‐1 and TSG101 (Figure S4b, Supporting Information), two classic EV markers that are primarily located inside vesicles.^[^ ^34^ ^]^ Eventually, 4974 and 4453 human proteins were identified in the surfaceome for CAM and T2AM EVs, respectively. Between the two datasets, 3475 proteins were shared, including reported (ARRDC1,^[^ ^35^ ^]^ PLXNA1,^[^ ^36^ ^]^ MFGE8,^[^ ^25^ ^]^ and GAPDH^[^ ^37^ ^]^; Figure 3b) and one in‐house tested (COPT1; Figure S5, Supporting Information) EV bioengineering scaffold proteins. Clathrin and ribosomal proteins were among the most abundant surface proteins (Figure S4c, Supporting Information). Next, we performed gene set enrichment analysis using the gene ontology (GO) database and found several significantly enriched GO terms describing vesicle biogenesis, cell binding, and hydrolytic activity on both EV types (Figure 3c).

To gain insight into subpopulation‐specific proteins, we applied differential analysis of CAM and T2AM EVs surfaceome. Aligning with our previous observations,^[^ ^33^ ^]^ TSPAN2 and CD63 were exclusively expressed on the EVs (Figure 3b). Of the 5952 surface proteins, 211 and 352 proteins were significantly (P ≤ 0.05, fold‐change ≥ 2) upregulated in CAM and T2AM EVs, respectively (Figure 3d). Moreover, the differentially expressed proteins were annotated through over‐representation analysis using the Reactome pathway database. Interestingly, T2AM‐EV‐specific surface proteins were significantly involved in the integrin cell surface interaction as well as extracellular matrix organization and degradation (Figure 3e). The core genes behind these pathways include extracellular matrix (ECM) components (collagen subunits, LAMA4 and FGG), ECM‐binding partners (ITGA1), and ECM‐processing‐related enzymes (ADAMTS1, CAPN15, CAST), and the TGFBR2 ligand SCUBE3. In contrast, CAM‐EV‐specific surface proteins might be involved in the attachment and/or entry of microorganisms (Figure 3e). While the precise mechanisms driving these differences require further investigation, our data suggest CD63 and TSPAN2 may mark distinct, potentially mutually exclusive EV subpopulations.

To confirm APEX2‐labeled proteins are expressed on EVs, we selected two example proteins, ITGA1 (integrin alpha‐1) and TUBB (tubulin beta chain), that were relatively abundant in our results and have commercially available fluorescent antibodies for the ease of detection. ITGA1 has transmembrane domains and is generally expressed on cell surfaces; whereas TUBB is the major constituent of microtubules and has no transmembrane domains and is classified as an intracellular protein. The EVs were stained with the antibodies and analyzed directly using imaging flow cytometry. Our results show that both CAM and T2AM EVs were positive for ITGA1 and TUBB (Figure S6, Supporting Information). Overall, the surface display of APEX2 using CD63 and TSPAN2 as scaffolds results in the efficient labeling of core surface proteins on EVs.

### 2.3 APEX2 Surface Labeling Identifies EV Corona Proteins

**Figure 4:**
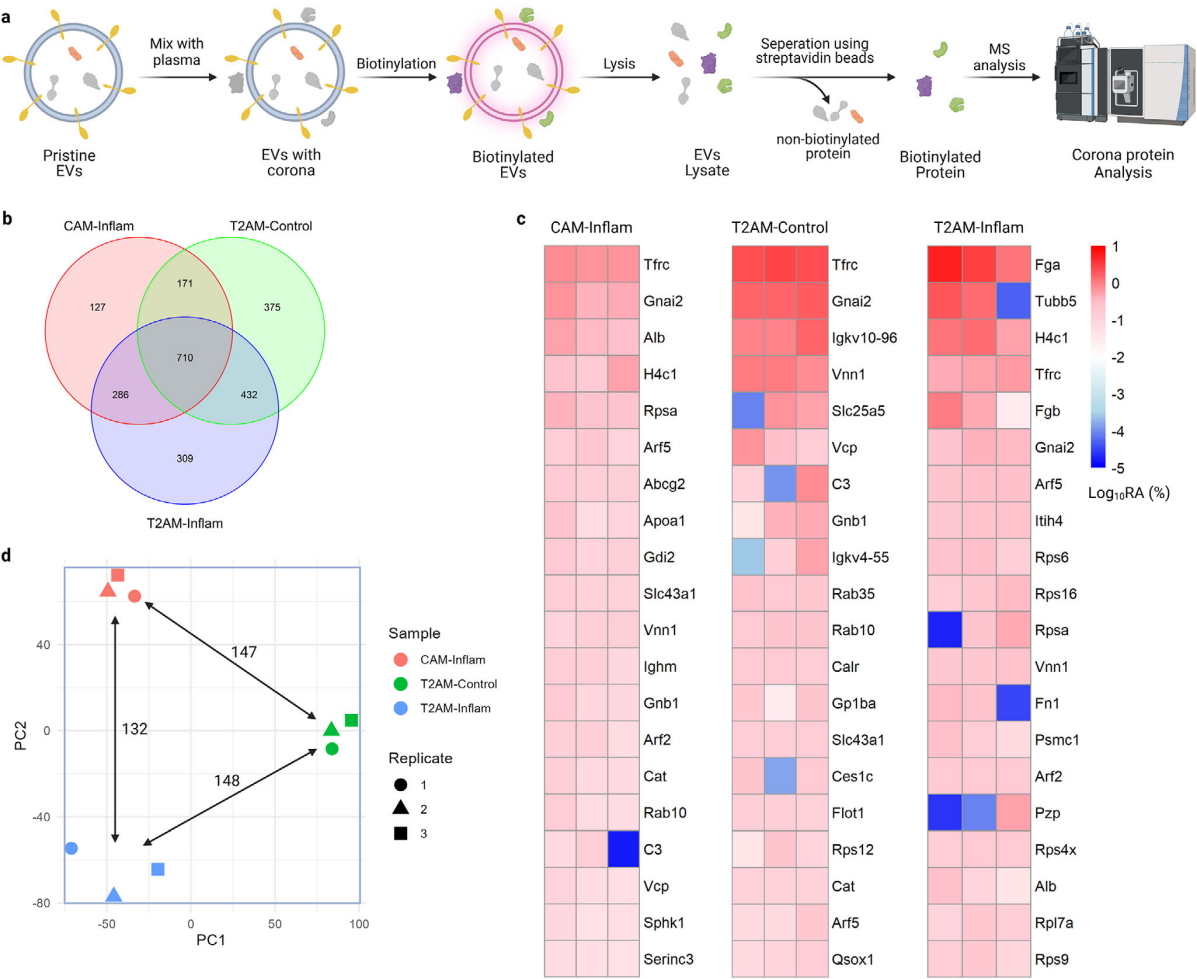
Proteomic analysis of vesicle corona after exposure to mouse plasma. a) EVs were mixed with mouse plasma and subjected to biotinylation. The mixture was lysed to release cargo proteins, and biotinylated proteins were enriched with streptavidin beads for proteomic analysis. b) Venn diagram of corona proteins identified in the three sample groups. c) Heatmap of relative protein abundance for each sample. RA: relative abundance. d) Principal component analysis of samples according to relative protein abundance. The value indicates centroid distance.

The EV corona has important biological functions, underscoring the significance of high‐fidelity resolution of corona proteins versus producer‐cell‐derived core surface proteins. To check whether vesicle surface APEX2 is functional in plasma, CAM and T2AM EVs were exposed to mouse plasma in vitro prior to biotinylation (**Figure** **4**a). Importantly, significant levels of biotinylation were detected (Figure S7a, Supporting Information), suggesting the maintenance of APEX2 activity. However, the detected labeling intensity in plasma was not as strong as in phosphate buffers. Given the high diffusibility of biotin‐phenol and hydrogen peroxide, the observed reduction in labeling efficiency was likely attributable to the limited diffusion of AF647‐streptavidin conjugates (≈60 kDa) within the corona layer.

Next, we collected plasma from healthy mice (control plasma) or mice with lipopolysaccharide‐induced systemic inflammation (inflamed plasma) and incubated CAM or T2AM EVs with the plasma for corona formation. Surprisingly, we found prominent levels of hemoglobin in all samples (relative abundance being 68–80%; Figure S7b, Supporting Information). To understand the underlying reasons, we assessed the level of hemolysis in the plasma used. Indeed, the plasma samples were visually yellowish and calorimetrically determined to have low level of hemolysis (1% relative to water treatment; Figure S7c, Supporting Information). On the other hand, hemoglobin has been reported to possess peroxidase activity^[^ ^38^ ^]^ and might thus confound APEX2‐dependent proximity labeling. For those reasons, we removed Hbb (hemoglobin subunit beta) and Hba (hemoglobin subunit alpha) from the identified list. Eventually, a total of 1688, 1737, and 1294 mouse proteins were identified in the T2AM‐Control, T2AM‐Inflam, and CAM‐Inflam samples, respectively, with 710 proteins being shared among them (Figure 4b). Transferrin receptor protein (Tfrc) was the most abundant corona protein on CAM‐Inflam and T2AM‐Control samples, while fibrinogen alpha chain (Fga) was the highest on T2AM‐Inflam sample (Figure 4c). Notably, although albumin and lipoproteins are commonly reported contaminants in corona analysis in other studies,^[^ ^18^, ^19^, ^20^ ^]^ they were not among the most abundant proteins in our datasets (Figure S7d,e Supporting Information), supporting the specificity of our approach.

Despite the commonality, we observed clear differences in the protein abundance among the samples (Figure S7f, Supporting Information). Principal component analysis of the corona proteins reveals three distinct clusters, which closely matched the known sample groupings (Figure 4d). Furthermore, EV subpopulation and matrix impose comparable effects on the corona composition according to cluster centroid distance (range: 132 to 148). Overall, the surface display of APEX2 effectively labels EV corona proteins after plasma exposure.

### 2.4 Pairwise Comparisons Reveal Subpopulation‐ and Matrix‐Dependent Corona Formation

**Figure 5:**
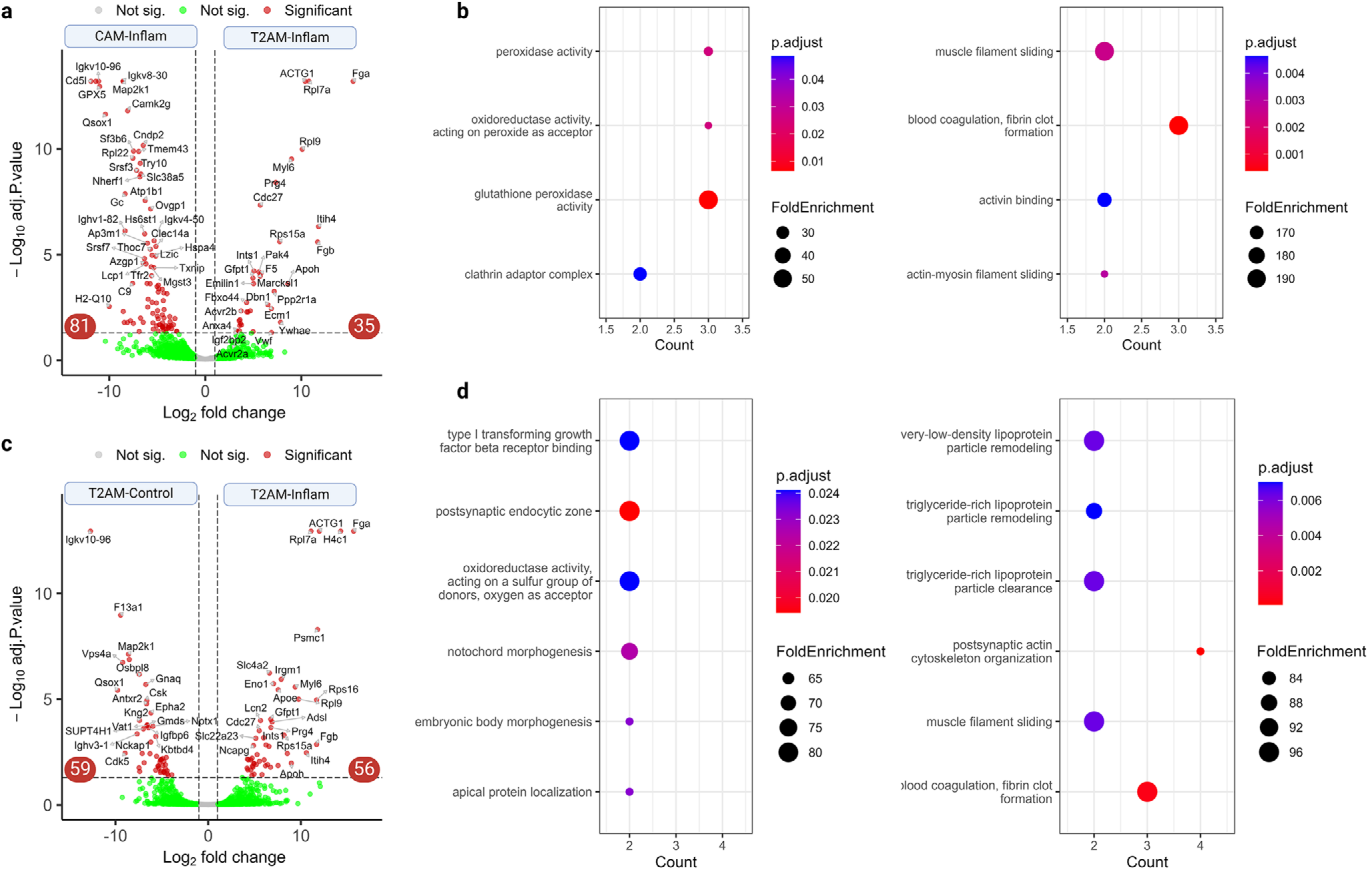
Vesicle subpopulation and matrix affect corona protein composition. a) Volcano plot of identified corona proteins of CAM and T2AM EVs after exposure to inflamed plasma. P ≤ 0.05 and fold‐change ≥ 2 considered significant. b) GO terms of upregulated proteins in CAM‐Inflamed (left) and T2AM‐Inflamed (right) samples. c) Volcano plot of identified corona proteins of T2AM EVs after exposure to control or inflamed plasma. P ≤ 0.05 and fold‐change ≥ 2 considered significant. d) GO terms of upregulated proteins in T2AM‐Control (left) and T2AM‐Inflamed (right) samples.

To understand the respective contribution of EV subpopulation and matrix type on corona composition, we performed pairwise differential expression analysis (P ≤ 0.05 and fold‐change ≥ 2 considered significant). A total of 116 proteins exhibited significantly differential expression patterns between T2AM and CAM EVs after exposure to inflamed plasma (**Figure** **5**a). Among the top upregulated corona proteins in T2AM‐Inflam samples were fibrinogen subunits (Fga and Fgb) and inter‐alpha‐trypsin inhibitor (Itih4); while CD5 antigen‐like (Cd5l), immunoglobulin kappa proteins (Igkv10‐96) and H‐2 class I histocompatibility antigen (H2‐Q10) were higher in CAM‐Inflam samples. GO enrichment analysis of differentially expressed proteins suggests that CAM‐Inflam samples were enriched with peroxidase activity (core gene: Gpx3/Gstp1/Mgst3) and clathrin adapter complex (core gene: Ap1m1/Ap3m1) effectors, while CAM‐Inflam samples were enriched with blood coagulation (core gene: Apoh/Fga/Fgb), activin binding (core gene: Acvr2a/Acvr2b), and filament sliding (core gene: Dbn1/Myl6) effectors (Figure 5b).

A similar comparison was conducted on T2AM EVs exposed to either control or inflamed plasma. The analysis shows that 56 and 59 proteins were significantly upregulated and downregulated in T2AM‐Inflam relative to T2AM‐Control, respectively (Figure 5c). More specifically, Fga, Histone H4 (H4c1) and 26S proteasome regulatory subunit 4 (Psmc1) were mostly upregulated on T2AM‐Inflam samples, while Igkv10‐96, vacuolar protein sorting‐associated protein 4A (Vps4a), and sulfhydryl oxidase 1 (Qsox1) were most downregulated. GO analysis of upregulated proteins in CAM‐Inflam reveals the enrichment of “response to lipopolysaccharide” effectors (p.adj = 0.036, FoldEnrichment = 5.06), including Akt1, Alad, Irgm1, Mapk3, and Rac1. Furthermore, Top GO terms in T2AM‐Inflam samples include lipoprotein particle processing (core gene: Apoe/Lipc), while control plasma exposure resulted in the enrichment with transforming growth factor beta receptor binding (core gene: Lrg1/Snx6) (Figure 5d). Taken together, the APEX2‐based protein probe can shed light on the effect of EV subpopulation and matrix on corona composition.

### 2.5 Proximity Labeling With APEX2 Identifies EV‐Interacting Proteins on Recipient Cells

**Figure 6:**
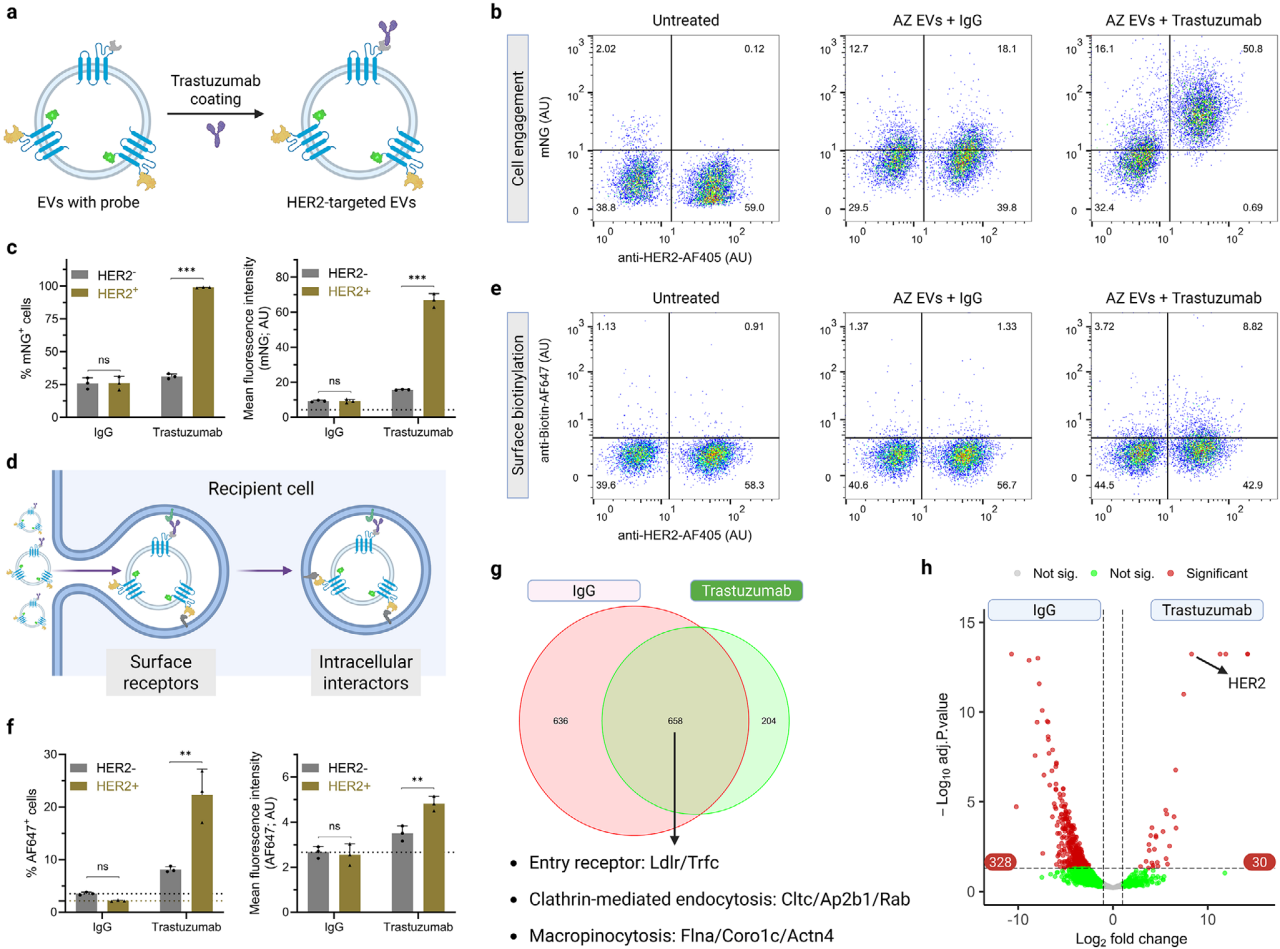
Vesicle surface APEX2 labels cellular interactors. a) Scheme of constructing HER2‐targeted EVs. HEK‐293T cells were transduced to co‐express TSPAN2‐APEX2‐mNG and TSPAN2‐ZZ (AZ cells), and the generated AZ EVs were exogenously coated with trastuzumab. b) Detection of mNG‐positive cells using flow cytometry. Recipient cells were a mixture of B16F10 cells with or without HER2 and treated with EVs for 2 h. c) Quantification of the fraction of mNG‐positive cells and mean fluorescence intensity in target (HER2^+^) and non‐target (HER2^−^) cells. The dash line indicates untreated cells. Mean ± standard deviation. Two‐tailed parametric unpaired *t* test. ns: non‐significant; ^***^: p ≤ 0.001. d) Scheme of EV‐cell interactions. During the process, vesicle surface APEX2 tags cell surface receptors and intracellular interactors. e) Detection of biotin‐positive cells using flow cytometry. Recipient cells were a mixture of B16F10 cells with or without HER2 and treated with EVs for 2 h and subjected to biotinylation before measurement. f) Quantification of the fraction of biotin‐positive cells and mean fluorescence intensity in target (HER2^+^) and non‐target (HER2^−^). Mean ± standard deviation. Two‐tailed parametric unpaired *t* test. ns: non‐significant; ^**^: p ≤ 0.01. g) Venn diagram showing the number of biotinylated proteins in recipient cells. h) Volcano plot of differentially expressed proteins. P ≤ 0.05 and fold‐change ≥ 2 considered significant.

Identifying cellular interactors is fundamental for elucidating cell‐dependent functions of EVs, such as contact‐dependent signaling and intercellular molecule exchange. To investigate whether vesicle‐surface APEX2 can label cellular receptors during EV–cell engagement, we designed a proof‐of‐concept experiment testing whether T2AM EVs could biotinylate predefined surface receptors on recipient cells. Our group has recently developed HER2 (human epidermal growth factor receptor 2)‐targeted EVs through endogenous surface display of the ZZ peptide (114 amino acids) that binds the Fc portion of the HER2 antibody trastuzumab.^[^ ^39^ ^]^ Here, the ZZ domain was cloned into the large extracellular loop of TSPAN2, yielding TSPAN2‐ZZ. To produce HER2‐targeted T2AM EVs, HEK‐T2AM cells were additionally transduced to stably express TSPAN2‐ZZ, and the EVs thereof (abbreviated as AZ) were exogenously coated with trastuzumab (**Figure** **6**a). Flow cytometry analysis reveals high colocalization of T2AM with TSPAN2‐ZZ fusion proteins at both cellular and single‐vesicle levels (Figure S8a, Supporting Information). Next, we treated a mixture of HER2‐negative and HER2‐positive (1:1) B16F10 cells with antibody‐coated EVs for 2 h and analyzed cellular mNG signals to confirm EV‐cell engagement (Figure S8b, Supporting Information). As expected, coating with trastuzumab, but not the IgG control antibody, significantly enhanced EV engagement with HER2‐positive cells relative to HER2‐negative cells (Figure 6b,c).

Next, to examine if vesicle surface APEX2 could biotinylate cellular interactors including surface receptors, we reused the co‐culture model and analyzed the extent of cell surface biotinylation (Figure 6d). The biotinylation assay (using cell‐impermeant streptavidin‐AF647 conjugates) provides a highly specific, instantaneous snapshot of EV engagement occurring exclusively at the cell surface during the strict 1‐min labeling window. In comparison to the mNG signals that reflect the cumulative result of EV uptake, we saw a small but appreciable fraction of HER2‐positive cells becoming AF647‐positive (Figure 6e). Notably, the trastuzumab coating resulted in a significant increase in cell surface biotin levels in HER2‐positive cells, with a 2.75‐fold higher fraction of stained cells and a 1.38‐fold higher intensity than in HER2‐negative cells (Figure 6f).

Furthermore, we used mass spectrometry to annotate the repertoire of biotinylated proteins. To better quantify recipient‐cell‐derived proteins in the presence of confounders like hemoglobin, EV proteins, and bovine serum proteins, we labeled HER2‐positive B16F10 cells with heavy amino acids (Arg_10_ and Lys_8_) using the SILAC (stable isotope labeling with amino acids in cultured cells) protocol. The cells were treated with isotype or trastuzumab‐coated AZ EVs for 2 h, subjected to biotinylation, and lysed to release protein contents for quantification (Figure S8c, Supporting Information). A technical negative control was included by omitting biotin‐phenol in the biotinylation step. This approach eventually identified 1294 and 862 heavy‐labeled proteins in the IgG and trastuzumab group, respectively (Figure 6g). A total of 658 proteins were shared between the two groups, which include commonly reported receptors for nanoparticle entry (e.g., low‐density lipoprotein receptor (Ldlr), Tfrc) and molecules involved in clathrin‐mediated endocytosis (e.g., clathrin heavy chain 1 (Cltc), AP‐2 complex subunit beta (Ap2b1), ras‐related proteins) as well as macropinocytosis (e.g., filamin‐A (Flna), coronin‐1C (Coro1c), alpha‐actinin‐4 (Actn4)).

In addition, we performed a differential expression analysis to gain insight of trastuzumab‐coating‐specific cellular interactors. A total of 30 and 328 proteins were upregulated and downregulated in the trastuzumab group, respectively (Figure 6h). To interpret these changes, we performed functional annotation of up/downregulated proteins using the Reactome database (Figure S9, Supporting Information). The analysis revealed that HER2 targeting led to a reduction of interactome components associated with RNA processing and SUMOylation pathways. Most importantly, HER2 was the most significantly upregulated interactor for trastuzumab‐coated EVs compared to IgG‐coated EVs (Figure 6h), suggesting that HER2‐directed targeting may shift the overall pattern of EV‐cell interactions toward a more selective binding profile. Taken together, vesicle surface APEX2 successfully tags the putative cell surface receptor.

## 3 Discussion

We have developed protein probes for high‐fidelity characterization of EV core surface proteins, corona proteins, and cellular interactors. Unlike corona proteins, which are acquired post‐secretion, EV core surface proteins originate from producer cells and are highly relevant for disease diagnosis.^[^ ^40^, ^41^ ^]^ However, their quantification in liquid biopsies remains challenging due to the low abundance of pathological‐cell‐derived EVs, particularly from hard‐to‐access tissues like the brain.^[^ ^40^ ^]^ In addressing this, our APEX2‐based probe enables specific tagging of EV core surface proteins for untargeted proteomic analysis. By expressing this probe in cells of interest, the EV core surface proteome can be resolved to facilitate biomarker discovery. Notably, our analysis of CD63‐ and TSPAN2‐engineered EVs reveals several reported bioengineering scaffold proteins within their core surface proteome. This finding highlights the potential for further data mining to identify novel scaffold proteins for EV bioengineering, as briefly explored for COPT1 (Figure S5, Supporting Information).

The EV corona contributes significantly to the overall biological effects of EVs,^[^ ^42^, ^43^ ^]^ underscoring the need for its specific characterization relative to core surface proteins. Corona proteins associate with EV surfaces through diverse mechanisms, and their characterization methods has employed various principles, including the use of lytic enzymes (trypsin, proteinase K, and RNase),^[^ ^44^, ^45^ ^]^ cation chelators (ethylenediaminetetraacetic acid) to disrupt electrostatic interactions,^[^ ^46^ ^]^ and reducing agents (tris(2‐carboxyethyl)phosphine) to cleave disulfide bonds.^[^ ^47^ ^]^ Proteins susceptible to these treatments are operationally defined as surface associated. Alternative approaches have relied on surface‐tethered proximity labeling enzymes or membrane‐impermeable chemical probes (like NHS‐biotin^[^ ^24^ ^]^ to label protein amines and aminooxy‐biotin^[^ ^48^ ^]^ to label glycoproteins). A critical step in these methods is the removal of confounders (such as free proteins and non‐vesicular particles) to yield a relatively pure batch of EVs. However, the purification process itself, particularly the shear force, might irreversibly deplete loosely associated corona proteins, potentially altering the native corona composition. To address this, we characterized plasma‐exposed EVs without prior purification, instead performing immediate biotinylation to capture a near‐native corona state. Unreacted biotin‐phenol was removed using gravity‐based size exclusion chromatography columns, minimizing the risk of biotinylated protein dissociation during processing. Thus, our protocol provides a benchmark for evaluating the efficiency of common isolation methods (e.g., polymer precipitation, ultracentrifugation, ultrafiltration) in preserving EV molecular signatures in biofluids.

Our study employed a cross‐species design, incubating human EVs with mouse plasma to quantify corona proteins, and confirmed that corona formation is dependent on the EV subset. The difference might be attributable to the distinct intrinsic surfaceome between CAM and T2AM EVs (Figure 3d), as variations in protein features, such as electrostatic properties, ligand‐binding domains, and structural motifs, likely influence plasma protein adsorption. However, due to the lack of cross‐species protein–interaction databases, further mechanistic interpretation is limited. To gain deeper mechanistic insights, future studies should use EVs and plasma from the same species and adopt strategies to distinguish EV‐ from plasma‐derived proteins. One option would be SILAC labeling of EV proteins prior to plasma incubation.

EVs affect recipient cells through two primary mechanisms: 1) contact‐dependent signaling without internalization; and 2) intracellular delivery of vesicular cargo molecules. Both mechanisms rely on the interactions between EV surface proteins and cell surface receptors at the interface. Therefore, comprehensively mapping EV surface proteins and their cellular interactors is essential to fully understand EV‐mediated cellular functions.^[^ ^41^ ^]^ Reported methods for identifying EV target cells primarily rely on quantifying the uptake of labeled EVs, which inherently overlook cells that respond solely via contact‐dependent signaling.^[^ ^22^ ^]^ In this study, we demonstrate that the APEX2‐based protein probe preferentially labels EV target cells over non‐target cells in vitro. This approach complements existing tools by enabling the detection of EV‐interacting cells regardless of internalization. Furthermore, in a proof‐of‐concept experiment, the probe specifically tags cell surface receptors, marking, to our knowledge, the first validation of major cellular interactors for EVs.

While our APEX2‐based probe offers significant advantages, several technical limitations should be noted. First, APEX2 was selected for its high reactivity in capturing transient EV‐cell interactions, but its reliance on hydrogen peroxide activation restricts in vivo applicability. Future work will explore alternative enzymes with comparable activity but improved biocompatibility. Second, high hemoglobin levels were detected in EV corona analyses. This is most probably an artefact due to the intrinsic peroxidase activity of hemoglobin,^[^ ^38^ ^]^ though the likelihood that EVs are super hubs for free hemoglobin might exist. Third, our comparative proteomic analysis relies on CD63 or TSPAN2 as scaffold proteins and targets only a subset of EVs expressing these tetraspanin proteins. While this strategy enables high‐fidelity surface display of APEX2, it inherently excludes CD63/TSPAN2‐negative EV subpopulations, a limitation shared by all scaffold‐dependent EV labeling methodologies. For comprehensive surfaceome and interactome mapping, further research might deploy new scaffolds for underrepresented EV subtypes and/or combine multiple scaffolds for surface display of APEX2 to expand coverage. Fourthly, tetraspanin proteins including CD9 and CD81 were reported to cluster as microdomains on EV surfaces, which might lead to spatially biased labeling. However, less is known for CD63 and TSPAN2 regarding their distribution on EV surfaces. Moreover, this limitation is partially mitigated by the effective labeling radius (≈20 nm) of APEX2, extending beyond immediate clustering sites.

Overall, the present study provides a novel framework for profiling EV surface proteins and mapping their interactions with environmental molecules and cells. The results are envisioned to enhance our understanding of EV biology and provide a foundation for developing EV‐based biomarkers and therapeutics.

## 4 Experimental Section

### Molecular Cloning

DNA sequences coding for proteins‐of‐interest were codon‐optimized for expression in human cells, ordered commercially (Integrated DNA Technologies and Twist Biosciences), and cloned downstream of the CAG promoter into the pLEX vector using restriction cloning strategies. Lentiviral transfer plasmids were synthesized by cutting transgenes from pLEX expression plasmids and inserting them into p2CL9IPwo5 backbone (plasmid was kindly given by H. Hanenberg, University Hospital Essen, Germany). All expression cassettes were confirmed by Sanger sequencing (Eurofins Genomics). Plasmids are available from the corresponding authors upon request.

### Cell Culture

HEK‐293T (ATCC, CRL‐3216) and B16F10 (ATCC, CRL‐6475) cells were maintained in high glucose DMEM (Gibco, 41966‐029) media supplemented with 10% fetal bovine plasma (FBS; Gibco, 10270‐106) and 1% anti‐anti (Gibco, 15 240). All cells were cultured in humidified incubators with 37 °C and 5% CO_2_. The cell cultures were mycoplasma‐free under routine tests.

### Lentivirus Production and Transduction

HEK‐293T cells were seeded in T‐175 flasks containing 20 mL DMEM and transfected upon confluency reaching ≈60%. Cells were co‐transfected overnight with 22 µg transfer plasmids, 22 µg helper plasmids pCD/NL‐BH, and 3.5 µg envelope plasmids pcoPE (encoding the human foamy virus envelope protein) that were pre‐complexed with 135 µg polyethyleneimine in 4 mL Opti‐MEM. The media was changed to full DMEM supplemented with 10 mM sodium butyrate (Sigma–Aldrich) for 8 h to induce gene expression and changed back to full DMEM. The conditioned media was collected 22 h later and filtered through 0.45 µm filters. The filtrate was spun at 25000 g for 90 min at 4 °C, and the pellet was suspended in 1 mL Iscove's modified Dulbecco's media supplemented with 20% FBS and 1% antibiotics‐antimycotics. The viruses were kept at ‐80 °C until use.

HEK‐293T cells were seeded in 6‐well plates until being ≈60% confluent and transduced with lentiviral particles overnight. To make stable cell pools, cells were passed a minimum of five times under antibiotics selection (4 µg mL^−1^ puromycin and/or 10 µg mL^−1^ blasticidin).

### Extracellular Vesicles Production

HEK‐293T‐derived stable cell lines were cultured in 15‐cm Petri dishes containing 20 mL DMEM until confluence reached ≈90%. Media was changed to Opti‐MEM and cells were cultured for an additional 48 h. The conditioned media were sequentially centrifuged (700 g for 5 min and 2000 g for 10 min) and filtered (0.2 µm) to deplete cells, cellular debris, and large particles. Then, the filtrate was diafiltrated and concentrated to roughly 80 mL using the KrosFlo KR2i TFF System (Repligen, US) with 300 kDa cut‐off hollow fiber filters (Spectrum Labs, D06‐E300‐05‐N) at a flow rate of 70 mL min^−1^ (transmembrane pressure at 3.0 psi and shear rate at 3700 sec^−1^). EVs were further concentrated till ≈0.5 mL using an Amicon Ultra‐15 spin‐filter with 10 kDa molecular weight cut‐off (Millipore, UFC901024) and stored at ‐80 °C in PBS‐HAT buffer until use.

### Nanoparticle Tracking Analysis

EVs were diluted in 200 nm‐filtered PBS if required and analyzed using ZetaView Twin (Particle Metrix) at the following settings: sensitivity of 75, shutter of 130, 11 position measurement scans per sample, 2 cycles. Particle size and concentration were derived using the default setting.

### Cellular and Vesicular Biotinylation

HEK‐293T‐derived stable cell and B16F10 cells were cultured in 24‐well plates containing 0.5 mL medium until confluency reached ≈60%. B16F10 cells were treated with 1e10 EVs for 2 h and gently rinsed with PBS twice. For the SILAC experiment, B16F10‐HER2 cells were maintained in arginine‐ and lysine‐free DMEM (Gibco) supplemented with 10% dialyzed FBS (Gibco) and heavy amino acids (0.398 mM Arg_10_ and 0.798 mM Lys_8_; Cambridge Isotope Laboratories) for at least five doubling times. The cells were cultured in 15‐cm Petri dishes, treated with 1e11 EVs for 2 h. To trigger cellular biotinylation, 5 µL biotin‐phenol (50 mM in DMSO, Sigma–Aldrich, SML2135‐50MG) was added per well and left for 30 min in the incubator. Then, 5 µL H_2_O_2_ (100 mM) was added and left for 1 min exactly at room temperature. Excess H_2_O_2_ was quenched using 0.5 mL antioxidant mixture (50 mM Trolox, 100 mM sodium ascorbate, 100 mM sodium azide). The cell viability was repeatedly more than 95% according to DAPI (4ʹ,6‐diamidino‐2‐phenylindole) staining.

Vesicular biotinylation was performed at a small scale. Briefly, EVs (5e10 particles in 200 µL) were incubated with a 200 µL matrix (PBS or plasma) in a tube at 37 °C for 30 min. Four µL biotin‐phenol (50 mM in DMSO) was added and incubated at 37 °C for another 30 min. Then, 4 µL H_2_O_2_ (100 mM in PBS) was added and left at room temperature (RT, 22 °C) for 5 min. Excess H_2_O_2_ was quenched using a 12 µL antioxidant mixture as before.

### Size Exclusion Chromatography

EVs were purified using gravity‐based size exclusion chromatography (SEC) columns (Izon, SP1) according to the manufacturer's instructions. The first 3 mL eluate was discarded as the void volume, while the next 2.5 mL eluate that was enriched with EVs was collected. For western blotting, the EV fraction was concentrated using 10 kDa cut‐off spin filters.

### Mouse Plasma Sampling

All mouse experiments were performed following the ethical permission granted by Swedish Jordbruksverket (permit No. 20281–2021). Balb/c mice (8‐week‐old) were bought from Charles River and housed in an animal facility for at least one week before use according to standard routines (ambient temperature: 20–22 °C, humidity: 45–55%, dark/light cycle: 12/12 h). Mice were intraperitoneally injected with lipid polysaccharide at 4 mg/kg. Blood was collected 5 h later into heparinized tubes and centrifuged at 2000 g for 10 min. The supernatant was collected if visually yellowish and stored at ‐80 °C until use.

### Western Blotting

EVs (2×10^9^ particles in 15 µL) were mixed with a 5 µL sample buffer (NP0007, Invitrogen) and heated at 70 °C for 10 min. The lysate was loaded onto a NuPAGE Novex 4–12% Bis‐Tris Protein Gel (Invitrogen, NP0335BOX) and separated in NuPAGE MES SDS running buffer (Invitrogen, NP0002) at 120 V for 2 h. Proteins were transferred to a nitrocellulose membrane (Invitrogen, IB23001) using the iBlot system. The membrane was immersed in blocking buffer (LI‐COR, 927–60004) for 1 h at room temperature and incubated with primary antibodies (1:1000 dilution for anti‐Calnexin [ThermoFisher, PA5‐19169], anti‐Syntenin‐1 [Origene, TA504796], Streptavidin‐680RD [926‐68079, LI‐COR]) overnight at 4 °C. Afterward, the membrane was rinsed using TBS supplemented with 0.1% Tween 20 (TBS‐T) for 3 times and stained with corresponding secondary antibodies (925‐68070, 926–32350, LI‐COR; 1:10000 dilution) for 1 h at RT. Eventually, the membrane was rinsed with TBS‐T 3 times, once with PBS, and visualized on the Odyssey infrared imaging system (LI‐COR, US).

### Streptavidin Pulldown

Biotinylated EVs were added to a lysis buffer containing 0.05% lauryl maltose neopentyl glycol (LMNG), 0.1% Triton‐X, and a protease inhibitor cocktail (Roche). Lauryl maltose neopentyl glycol (LMNG) was added to prevent protein adsorption to the tube.^[^ ^49^ ^]^ The samples were further sonicated using Bioruptor for 3 min with a 15‐sec run and 15‐sec intervals. To pull down biotinylated proteins, the EV lysate was incubated with 20 µL of streptavidin magnetic beads (Pierce, 88 817) at 4 °C for 2 h under rotation. Protein‐bound beads were sequentially rinsed with RIPA buffer (once), 1 M KCl solution (once), 0.1 M Na_2_CO_3_ solution (once), and 50 mM NH_4_HCO_3_ buffer (twice). For every rinse step, the beads were well suspended in a 1 mL buffer and rotated for 1 min before separating on a magnetic stand.

To liberate proteins for proteomics analysis, the beads were suspended in 200 µL 50 mM NH_4_HCO_3_ buffer supplemented with 0.02% LMNG. Then, proteins were reduced and alkylated with 10 mM tris(2‐carboxyethyl)phosphine and 50 mM 1 M chloroacetamide at 37 °C for 30 min. Proteins were digested using 0.5 µg trypsin (Promega) at 37 °C overnight under shaking (>1000 RPM). The next day, the supernatant was transferred to new tubes and acidified with 20 µL 10% trifluoroacetic acid. Peptide samples were desalted using the SDB‐RPS StageTip.^[^ ^50^ ^]^

### LC/MS/MS Analysis

LC/MS/MS analysis was performed on an Orbitrap Eclipse mass spectrometer (Thermo Fisher Scientific) equipped with a FAIMSpro interface, combined with a Vanquish Neo UHPLC pump (ThermoFisher Scientific). The mobile phases consisted of (A) 0.1% formic acid and (B) 0.1% formic acid and 80% acetonitrile (ACN). Peptides were loaded on a self‐made 18 cm fused‐silica emitter (100 µm inner diameter) packed with ReproSil‐Pur C18‐AQ (1.9 µm, Dr. Maisch, Ammerbuch, Germany), and separated by a linear gradient for 60 min (5–40% B over 45 min, 40–99% B over 5 min, and 99% B for 10 min) at the flow rate of 250 nl/min. FAIMS compensation voltages (CVs) were fixed to –45. MS scanning was performed in the data‐dependent acquisition (DDA; for SILAC experiment) or data‐independent acquisition (DIA; for other experiments) mode using the Orbitrap analyzer. MS1 scans were performed in the range of 350 to 1000 *m/z* (resolution = 120000, maximum injection time = 45 ms, and automatic gain control = 300%). In the following MS/MS scans, the precursor range was set to 500 to 740 *m/z*, and 20 scans were acquired with the isolation window of 12 *m/z*, with HCD normalized collision energy of 27 (resolution = 50000, injection time = 86 ms, auto gain control = 1000%, first mass = 120 m/z).

Raw spectra files were processed with default settings using DIA‐NN or DDA (v.1.8.1)^[^ ^51^ ^]^ to perform a library‐free search against the UniProt/SwissProt human and/or mouse database (downloaded from UniProt February 2024) combined with the contaminant database obtained from MaxQuant.^[^ ^52^ ^]^ The following additional options were used: protein‐qvalue 0.01; peak‐translation; mass‐acc‐cal 10; relaxed‐prot‐inf; matrix‐qvalue 0.01; matrix‐spec‐q; and top 4. The proteomics data are deposited to the ProteomeXchange Consortium via jPOST partner repository with the dataset identifier JPST003915 (PXD065777 for ProteomeXchange).^[^ ^53^ ^]^

Data was primarily analyzed using the DEP package (version 1.28.0). Briefly, LFQ intensity was log‐transformed, normalized using the variance stabilizing transformation model, and background‐subtracted from non‐biotinylated samples. Proteins with at least one valid value in any group were kept for further analysis. Hemoglobin was reported to have intrinsic peroxidase activity^[^ ^38^ ^]^ and might confound the EV surface‐restricted activity of APEX2; thus, it was removed from the curated protein list. Missing values were imputed by a normal distribution with a downshift of 1.8 standard deviations (SDs) and a width of 0.3 SDs. Relative abundance for individual protein was presented as the percentage of its intensity to the sum of intensity for all proteins in each sample. Significantly differentially expressed proteins were defined to have *adj*.P.value ≤ 0.05 and fold‐change ≧ 2. Gene set enrichment analysis and over‐representation analysis were conducted using the clusterProfiler package (version 4.14.6).^[^ ^54^ ^]^ All analyses were conducted using R (version 4.4.3).

### Flow Cytometry

Cell surface biotin was stained with 100 µL streptavidin‐AF647 conjugates (10 ng mL^−1^) or anti‐HER2 fluorescent antibodies (clone#511; Sino Biological) at room temperature for 30 min. After trypsin or Acctuase treatment, cells were resuspended in 100 µL of PBS containing 2% FBS. DAPI staining was applied to gate out dead cells. The samples were measured with a MACSQuant Analyzer 16 cytometer (Miltenyi, Germany). Data were analyzed with FlowJo software (version 10.6.2), and doublets were excluded by forward scatter area versus height gating.

EVs were analyzed using imaging flow cytometry (Amnis CellStream, Luminex) which quantifies fluorescently labeled particles. 25 µL EVs (maximum concentration 1 × 10^10^ particles/mL) were stained with 5 µL antibodies at room temperature overnight and diluted by 200‐fold in PBS‐HAT buffer. Samples were measured with FSC turned off, SSC laser set to 40%, and all other lasers (405, 488, 561, and 642 nm) set to 100%. EVs were defined as SSC (low) by using mNG‐tagged EVs as biological reference material, and regions to quantify mNG^+^ or AF647^+^ fluorescent events were set according to unstained non‐fluorescent samples and single fluorescence positive mNG‐tagged reference EV controls. Data was analyzed using FlowJo software (version 10.6.2). The following antibodies and concentrations were used: streptavidin‐AF647 conjugate (6 ng µL^−1^; ThermoFisher; S21374), APC‐isotype antibody (10 ng µL^−1^; Miltenyi Biotec; 130‐113‐434), and anti‐CD49a‐AF647 (20 ng µL^−1^; Nordic Biosite; 328 310). Gating strategy for cells and EVs are available in Figure S10 (Supporting Information). Light scatter detection of un‐labeled EVs is suboptimal for the instrument/settings used; therefore, quantification is reliant on fluorescently labeled EVs.

### Statistics and Reproducibility

Results are shown as mean (± SD) of biological replicates. The two‐sided Student's *T* test and one‐way ANOVA were used to compare the mean difference between two or more groups, respectively. No statistical method was used to predetermine the sample size. No data was excluded from the analysis unless otherwise indicated. The Investigators were not blinded to allocation during experiments and outcome assessment.

## Conflict of Interest

SEA is a consultant for and has equity interests in EVOX Therapeutics Ltd., Oxford, UK. The other authors declare no competing interests.

## Author Contributions

WZ conceptualized the study, performed experiments and bioinformatic analysis, and wrote the manuscript. MM performed experiment and bioinformatic analysis. WH performed experiments. KI designed and performed the proteomic analysis. SEA and DWH supervised the project and provided funding. All authors edited the manuscript before submission.

## Supporting information



Supporting Information

## Data Availability

The data that support the findings of this study are available from the corresponding author upon reasonable request.
