# Supplementary material for: Development of Endogenous Protein Probes for Characterizing Surface Proteins and Cellular Interactors of Extracellular Vesicles
Source: Adv Sci (Weinh). 2025 Dec 12;13(12):e11414. doi: 10.1002/advs.202511414 (PMC12948232; doi:10.1002/advs.202511414)
Supplement: Supplementary file 1 — Supporting Information [file ADVS-13-e11414-s001.docx]

**Development of endogenous protein probes for characterizing surface proteins and cellular interactors of extracellular vesicles**

Wenyi Zheng^1,2,3,4, *^, Metoboroghene Mowoe^2,3,4^, Wenqing Hou^2,3,4^, Daniel W. Hagey^2,3^, Koshi Imami^5, *^, Samir EL Andaloussi^2,3,,4 *^

1. College of Pharmacy, Chongqing Medical University, 400016 Chongqing, China

2. Division of Biomolecular and Cellular Medicine, Department of Laboratory Medicine, Karolinska Institutet, 14152 Huddinge, Stockholm, Sweden

3. Department of Cellular Therapy and Allogeneic Stem Cell Transplantation (CAST), Karolinska University Hospital, 141 86 Stockholm, Sweden

4. Karolinska ATMP Center, Karolinska Institutet, 14152 Huddinge, Stockholm, Sweden

5. RIKEN Center for Integrative Medical Sciences, Yokohama City, Kanagawa,

230-0045, Japan

*. Correspondence: [wenyi.zheng@cqmu.edu.cn](mailto:wenyi.zheng@cqmu.edu.cn); [koshi.imami@riken.jp](mailto:koshi.imami@riken.jp); [Samir.el-andaloussi@ki.se](mailto:Samir.el-andaloussi@ki.se)


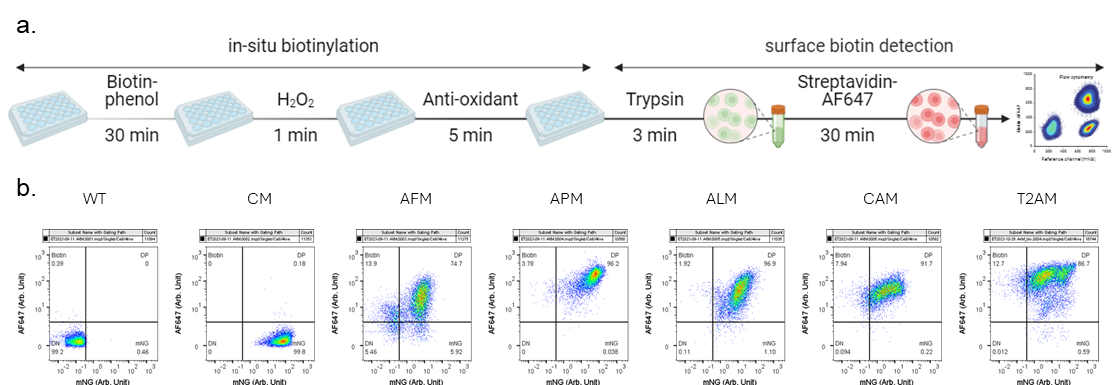
**Figure S1. Validation of APEX2 activity on cell surface.** (a) Cells were grown in 24-well plates and treated with biotin-phenol for 30 min. Biotinylation was triggered via a short treatment with H_2_O_2_ and quenched with antioxidants. Afterwards, cells were trypsinized and stained with streptavidin-AF647 conjugates before flow cytometry analysis. Dead cells were gated out using DAPI. (b) Detection of APEX2 construct expression and activity in cells. The parent gate was single live cells.

**
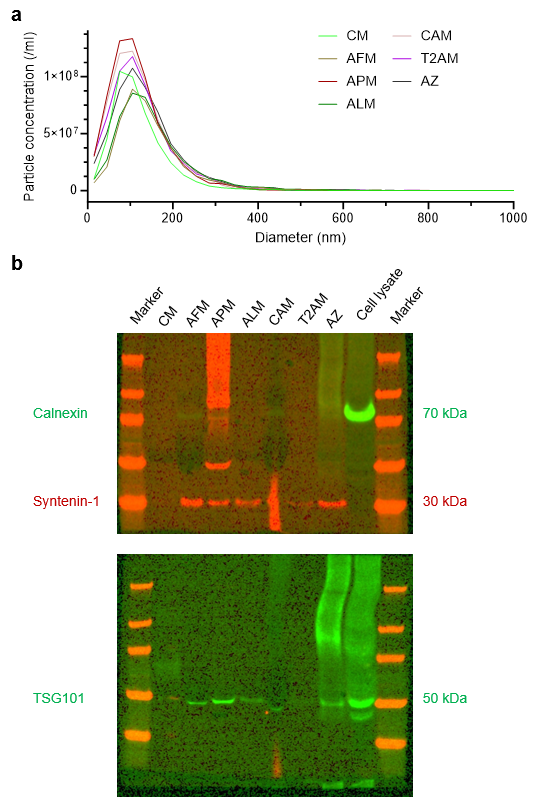
Figure S2. Physiochemical characterization of extracellular vesicles.** (a) Size distribution analysis using ZetaView. (b) Blots against common vesicle markers including Syntenin-1 and TSG101 and the exclusion marker Calnexin.


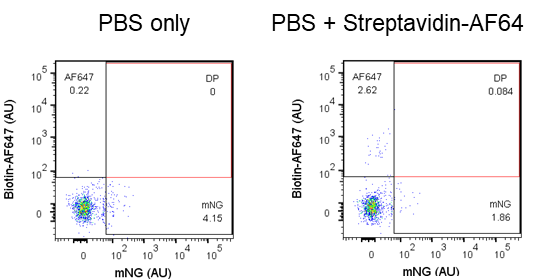
**Figure S3. Detection of streptavidin conjugates using imaging flow cytometry.** PBS was mixed with streptavidin-AF647 conjugates and analyzed using CellStream flow cytometer.

**
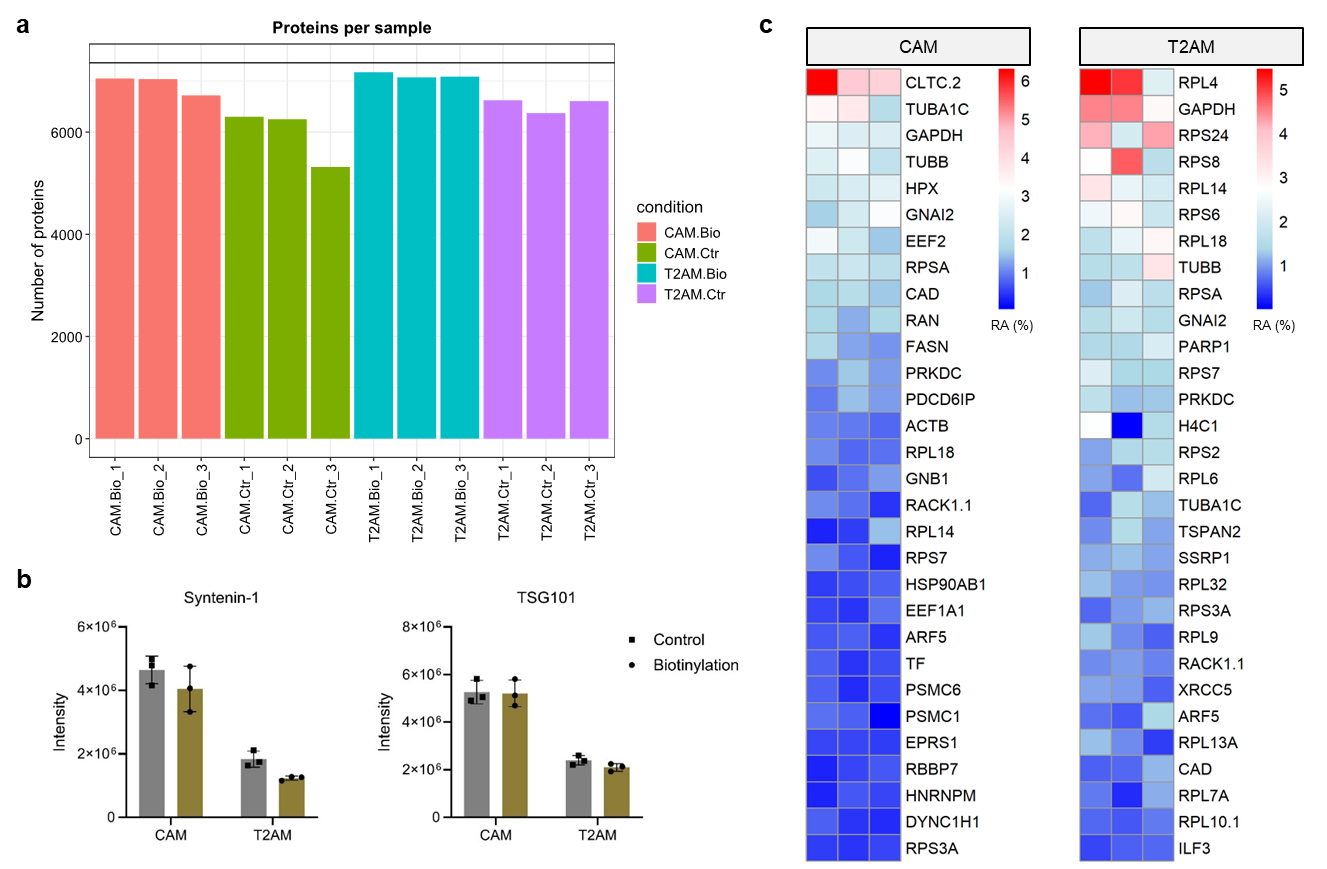
Figure S4. Proteomic analysis of core surface proteins on extracellular vesicles.** (a) Total number of proteins before applying background correction. (b) Intensity of Syntenin-1 and TSG101 retrieved from mass spectrometry analysis. (c) Heatmap showing the relative abundance of top 20 proteins. Relative abundance (RA) was presented as the percentage of the intensity for individual protein to the sum of intensity for all proteins.

**Figure S5. Exploration of COPT1 as a bioengineering scaffold.** (a) COPT1 (high-affinity copper uptake protein 1) was selected from the EV core surface proteome based on a few criteria: (i) molecular weight <50 kDa to facilitate protein expression; (ii) presence of at least one transmembrane domain to ensure stable membrane anchoring; (iii) an extracellular terminal for surface display; and (iv) an intracellular terminal for luminal loading. As proof-of-concept, the ZZ domain (for binding antibody Fc regions) and mNG reporter were fused at its N-terminus and C-terminus, respectively. (b-c) HEK293 cells transfected with ZZ-COPT1-mNG constructs for EV production. The cells and EVs were stained with VioBlue-conjugated isotype antibodies and analyzed using imaging flow cytometry.
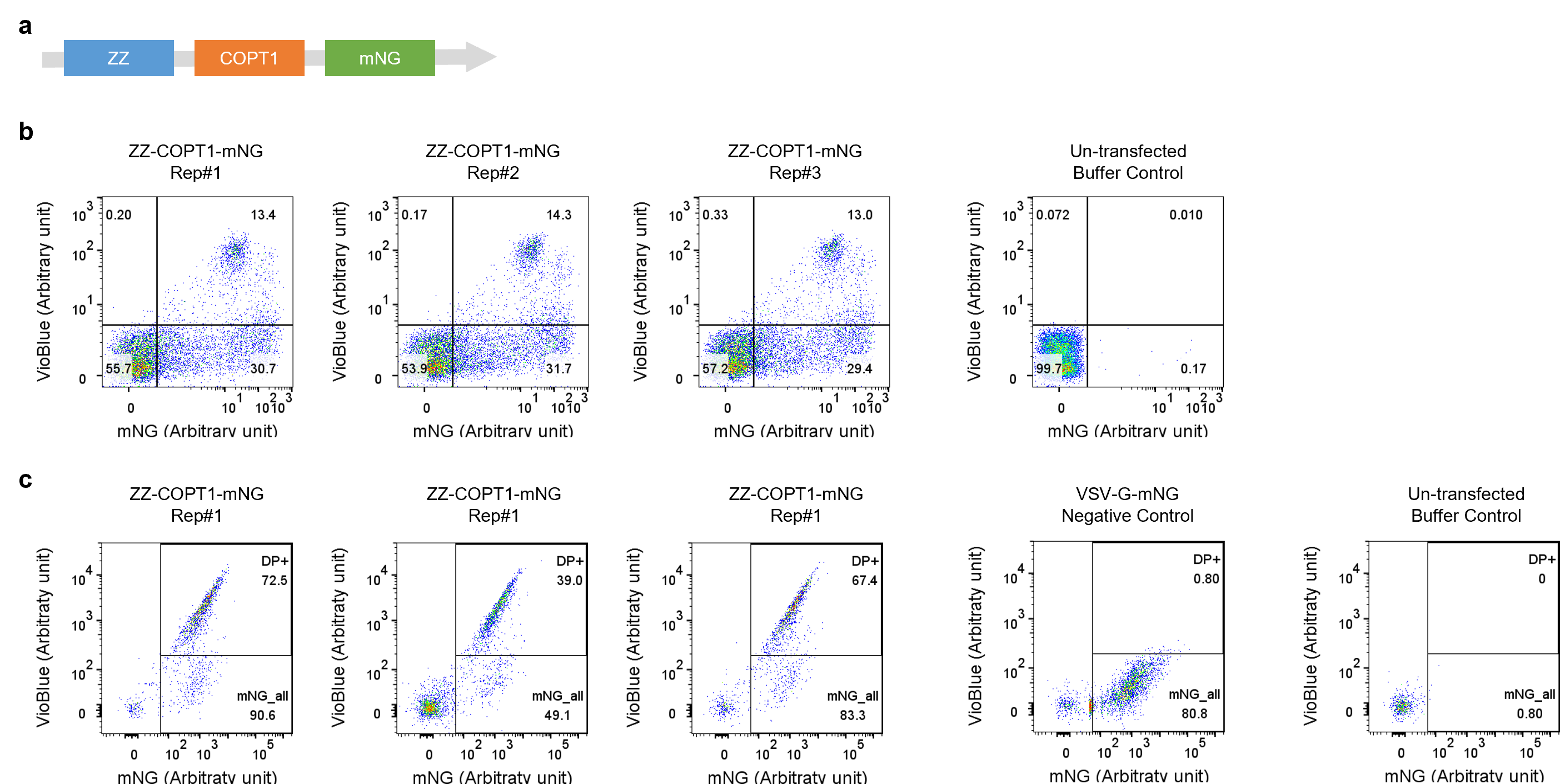


**
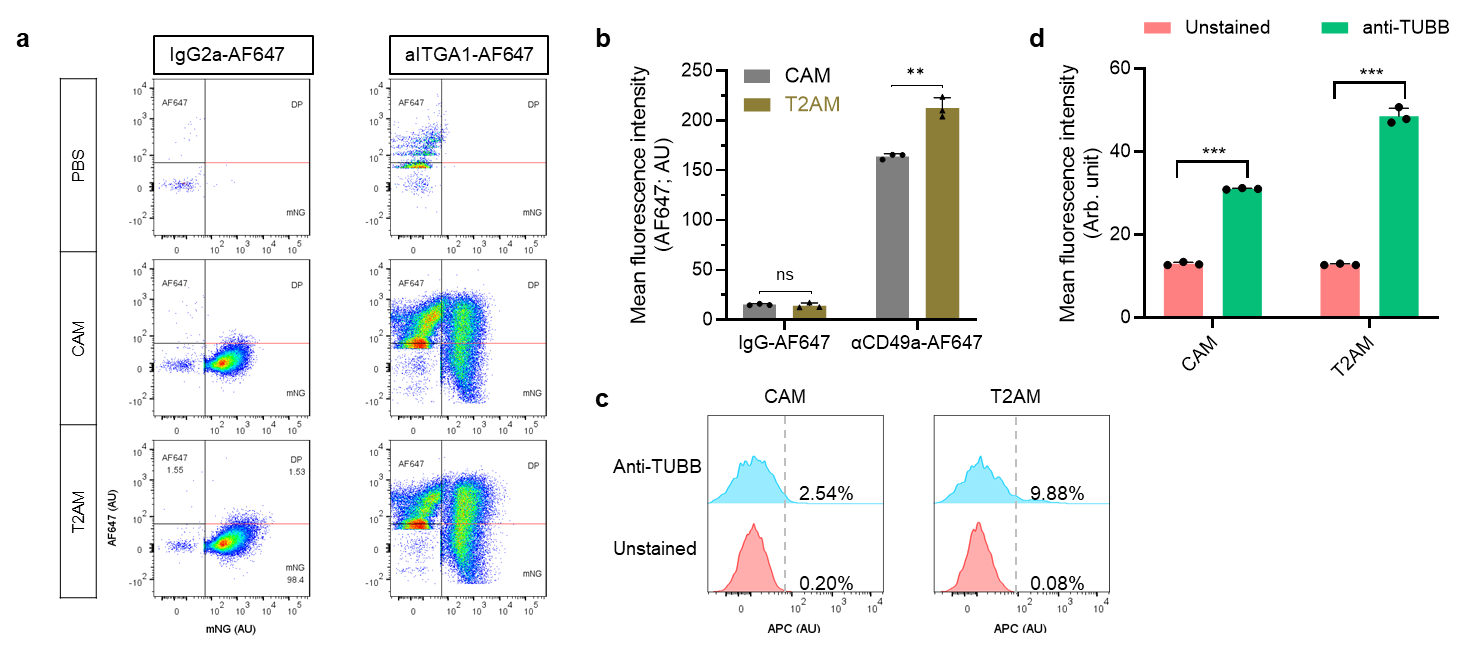
Figure S6. Flow cytometric analysis of core surface proteins on extracellular vesicles.** (a) Detection of ITGA1 protein on single vesicles using imaging flow cytometry. (b) Quantification of mean ITGA1 expression on mNG-positive populations. (c) Detection of TUBB protein on single vesicles using imaging flow cytometry. (d) Quantification of mean TUBB expression on mNG-positive populations. In panel (b&d), results were presented as mean ± standard deviation. Two-tailed parametric unpaired *t* test. ns: non-significant; ***: p ≤ 0.001.

**
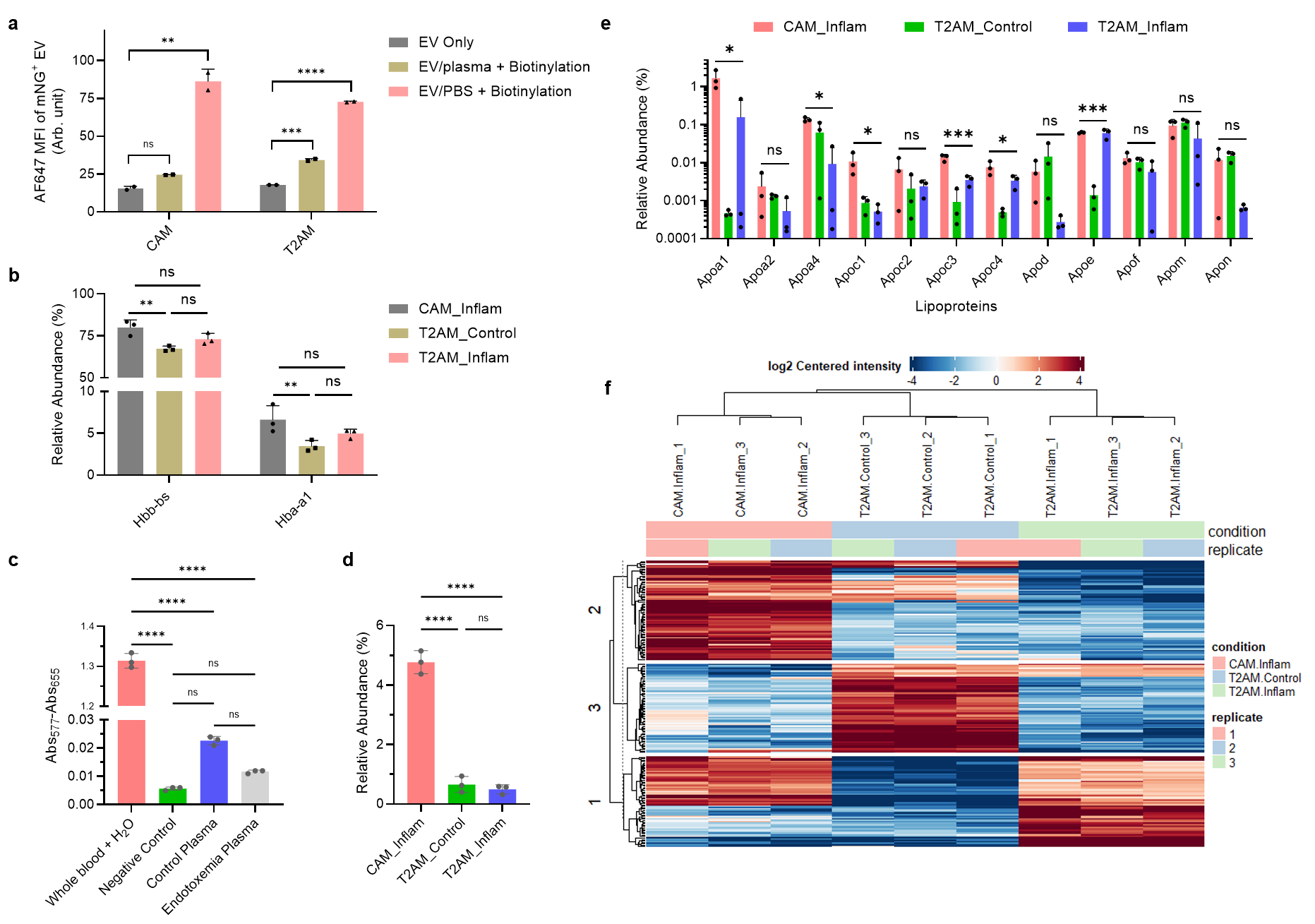
Figure S7. Analysis of vesicle corona proteins after exposure to mouse plasma.** (a) Analysis of vesicle surface APEX2 activity in nascent and plasma-exposed EVs. (b) Relative hemoglobin abundance in the samples. Relative abundance (RA) was presented as the percentage of the intensity for individual protein to the sum of intensity for all proteins. (c) Relative hemolysis level of plasma samples. (d) Relative mouse albumin abundance in the samples after removing hemoglobin. For (a-d), two-tailed parametric unpaired *t* test. (e) Relative lipoproteins abundance in the samples after removing hemoglobin. One-way ANOVA. (f) Heatmap of biotinylated proteins in plasma-exposed EVs. For all panels, data are shown as the mean ± standard deviation of biological replicates. ns: non-significant; *: p ≤ 0.05; ***: p ≤ 0.001.



**Figure S8. Flow cytometric analysis of cellular interactors of extracellular vesicles.** (a) Quantification of the colocalization of T2AM and TSPAN2-ZZ fusion proteins on cells and EVs. (b) Scheme of detecting surface biotin levels on recipient cells using flow cytometry. Recipient cells were a mixture (1:1) of B16F10 cells with or without HER2 expression and treated with EVs for 2 h. Subsequently, cells were biotinylated and stained with streptavidin-AF647 and anti-HER-AF405 conjugates for flow cytometry analysis. (c) Scheme of detecting biotinylated proteins in recipient cells using mass spectrometry. Recipient cells were HER2-expressing B16F10 cells and labeled with heavy isotopes. Following EVs treatment for 2 h, the cells were subjected to biotinylation and lysed. Afterwards, biotinylated proteins were enriched with streptavidin beads and quantified using mass spectrometry.


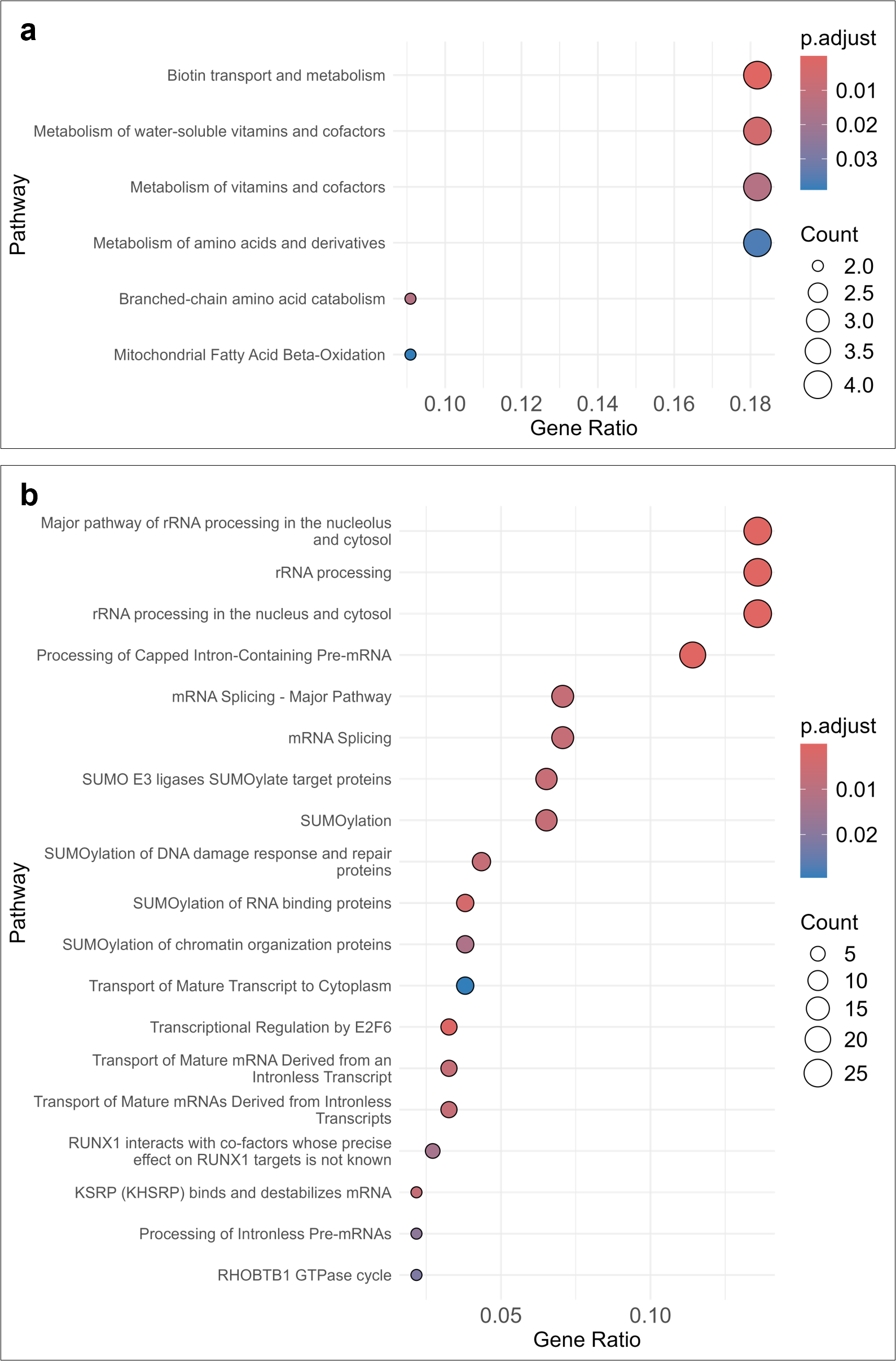
**Figure S9. Pathway analysis of differentially expressed mouse genes in HER2-targeted group.** (a) Upregulated genes. (b) Downregulated genes. All significant pathways were shown. p ≤ 0.05, False Discovery Rate (FDR) ≤ 0.2 (Benjamini–Hochberg).


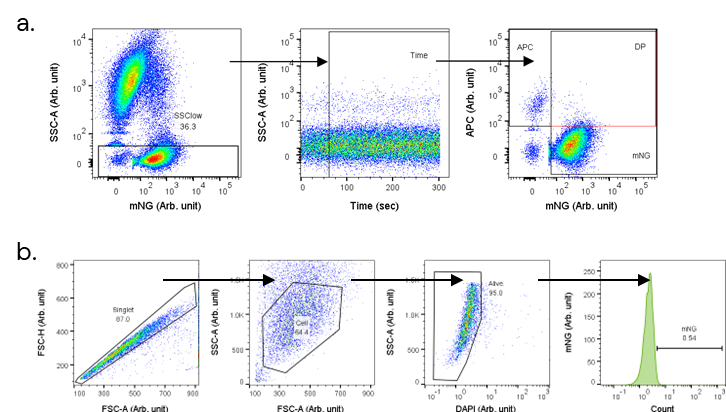
**Figure S10. Gating strategy for extracellular vesicles (a) and cells (b) in flow cytometry.**
